# *Bordetella pertussis*, Finland and France

**DOI:** 10.3201/eid1206.051283

**Published:** 2006-06

**Authors:** Valérie Caro, Annika Elomaa, Delphine Brun, Jussi Mertsola, Qiushui He, Nicole Guiso

**Affiliations:** * Institut Pasteur, FRE-CNRS 2849, Paris, France;; † National Public Health Institute and University of Turku, Turku, Finland

**Keywords:** Bordetella pertussis, pulsed-field gel electrophoresis, genotyping, Finland, France, dispatch

## Abstract

We used pulsed-field gel electrophoresis analysis and genotyping to compare clinical isolates of *Bordetella pertussis* recovered since the early 1990s in Finland and France, 2 countries with similar histories of long-term mass vaccination with whole-cell pertussis vaccines. Isolates from both countries were similar genetically but varied temporally.

The introduction of whole-cell pertussis vaccines (wP) from the 1940s to the 1960s in many countries resulted in a dramatic decrease in illness and death from pertussis. However, pertussis remains a considerable public health problem worldwide. Indeed, the disease continues to be endemic in vaccinated populations in Europe, Australia, Canada, and the United States, with cyclic increases at 3- to 5-year intervals, despite high vaccination coverage (1,2). One of the major causes is waning of vaccine-induced immunity with time (1,3), but increased disease surveillance and use of biologic diagnosis are also implicated. However, changes in the agent of the disease, *Bordetella pertussis*, are of some concern. This bacterium expresses adhesins such as filamentous hemagglutinin, pertactin, fimbriae (FIM), and toxins such as pertussis toxin, and adenylate cyclase-hemolysin (4). Recently, circulating isolates were found to differ from the strains used for the wP vaccines in the world (5–13). These observed changes might modify the properties of the isolates and affect the efficacy of pertussis vaccines.

In Europe, heterogeneity is high in epidemic situations with respect to wP vaccines used and vaccination history and strategy. However, Finland and France have implemented similar mass wP vaccination programs with high coverage for several decades. Since 1952, children in Finland have been vaccinated with combined diphtheria-tetanus wP vaccine (DTwP) at 3, 4, and 5 months and from 20 to 24 months of age. The wP vaccine contains 2 strains and has remained unchanged since 1976. Since 1959, children in France have been vaccinated with DTwP-inactivated polio vaccine at 3, 4, and 5 months and from 16 to 18 months of age. The vaccine calendar for primary vaccination was changed to 2, 3, and 4 months of age in 1995. The same wP vaccine, composed of 2 strains, has been used for >40 years. In both Finland and France, incidence of pertussis has increased and the disease has shifted to older age groups, especially adolescents and adults. The cycles of pertussis disease are observed every 3–5 years (5,13). The aim of this study was to analyze and compare the isolates circulating in the 2 European countries with long-term and intensive vaccination.

*B. pertussis* isolates were selected from collections of the Finnish Pertussis Reference Laboratory of the National Public Health Institute (Turku, Finland) and the French Pertussis National Center of Reference (Paris, France). Of the 503 Finnish isolates recovered from 1991 to 2004, 64 were selected either because they represent all available isolates from 1 community (Paimio) or they were recovered from a geographic area as wide as possible. In addition, 6 isolates from a school outbreak that occurred in Heinavesi (Finland) in 1982 were included to study the changes over time. Of the 1,049 French isolates recovered from 1991 to 2004, we selected 61 because they are representative of the French collection from a temporal and geographic viewpoint and they correlate with the different cycles of pertussis observed in France and Finland.

All Finnish and French isolates showed a high similarity with a minimum of 80.3% overall relatedness by using pulsed-field gel electrophoresis (PFGE) analysis after digestion of *B. pertussis* genomic DNA with *Xba*I restriction enzyme (14) (Figure A1). Most Finnish and French isolates fell into PFGE groups III, IV, and V (Figure A1 and Figure , panel A) corresponding to the isolates circulating in Europe from 1999 to 2001 (14). A new PFGE group (VII) was identified among Finnish isolates recovered in 2004 (Figure A1 and Figure , panel A). This new profile, with 97.3% level of relatedness (Figure A1), might represent an emerging group. The new profile was further confirmed with PFGE by using the second restriction endonuclease *Spe*I (data not shown), as previously recommended (5,14). We cannot say whether PFGE VII isolates are actually emerging or whether evidence for their emergence is anecdotal, as is the case with French PFGE VI isolates, which represent only 0.6% of French isolates (data not shown).

We previously showed that the major PFGE group circulating in Europe from 1999 to 2001 is group IV (14). In France and in Finland, the PFGE group IV was overrepresented from 1992 to 2004 with 85.3% level of relatedness, confirming the limited polymorphism of *B. pertussis*. PFGE group IV was subdivided into 3 subgroups, α, β, and γ, whose frequencies varied between countries (14). In our study, we show that, as in France (5) the PFGE groups of the isolates circulating in Finland vary temporally with the cycles of the disease (Figure A1 and Figure , Panel A). However, the frequency of the isolate subgroups circulating in Finland and France was different. In fact, the major subgroup detected in Finland between 1992 and 1999 was IVγ; since 2000, subgroups IVα and IVβ have been found (Figure , panel A). This circumstance is well illustrated among the isolates recovered in Paimio, where 99% of isolates with IVγ were circulating in 1992 whereas in 2004, 42% of subgroup IVβ, 33% of subgroup IVα, and 25% of the new group VII and none of IVγ were circulating in Paimio. Subgroups IVα and β, absent from 1992 to 1994, are now circulating. However, subgroup IVα is not a new subgroup in Finland since the 6 isolates collected in Heinavesi in 1982 exhibit this profile (Figure A1).

The analysis also included genotyping of the genes encoding pertussis toxin S1 subunit (*ptxA*) and pertactin (*prn*) and serotyping of FIM, performed as described previously (5,13). The sequence of *ptxA* is the same (*ptxA* allele type 1) for all Finnish and French isolates. The same types of *prn* alleles are also harbored by Finnish and French isolates (Figure A1). The emergence of isolates harboring *ptxA1* and *prn2* or *prn3* alleles in both countries might be explained by the fact that the wP vaccine strains used in both countries harbor *ptxA2* or *A4* and *prn1* alleles (13,15). A similar hypothesis might be proposed for the expression of FIM. In fact, most of the Finnish isolates collected from 1991 to 2004 express FIM2, whereas in France most of the isolates express FIM3 (Figure , panel B). The differences in the expression of FIM between Finnish and French isolates might reflect the difference in strains used for Finnish and French wP vaccines. The Finnish wP vaccine contains 2 strains expressing FIM2,3 and FIM3, whereas the French wP vaccine includes 2 strains expressing FIM2,3 and FIM2 (13,15). A marked shift of predominant serotype from FIM2 to FIM3 has been observed in Finland since 1999, although the wP vaccine remained the same. The emergence of isolates with FIM3 and PFGE subgroup IVβ, a new subgroup found in Finland, might be due to the increase in the frequency of this subgroup in the neighboring countries and the increased mobility of people within the European Union in the last decades.

We show that the *B. pertussis* isolates circulating in 2 countries with a long history of wP vaccination are genetically close. In the 2 countries, similar PFGE groups and subgroups are present, but their frequencies were different in the 1990s. Further, the subgroup emerging according to the cycles of pertussis in each country varies. The difference observed in frequency of subgroups could be due to herd immunity or human density of the populations concerned. Does this herd immunity vary depending on the human genetic population concerned or are the vaccine strains used expressing similar factors but not at the same level (e.g., FIM2 vs. FIM3)? This question needs further investigation.

These 2 countries have started using acellular pertussis vaccines, France since 2002 and Finland since 2005. Continued monitoring of the circulating isolates will be important.

**Figure:**
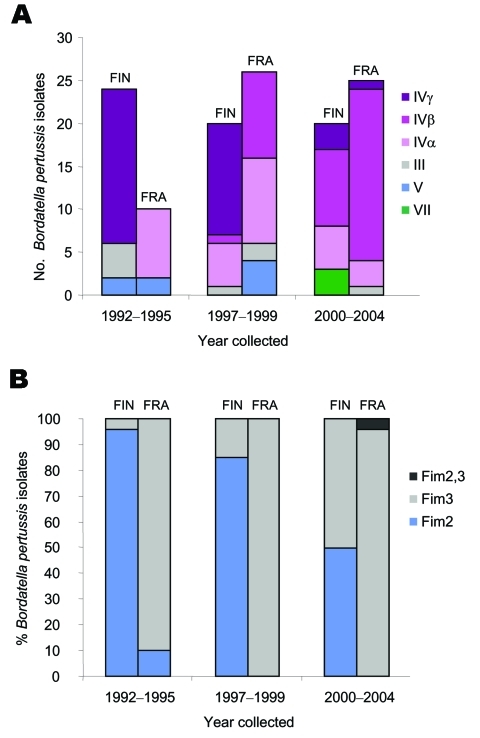
A) Pulsed-field gel electrophoresis profile repartition of *Bordetella pertussis* isolates by year and by country. B) Fimbriae expression of *B. pertussis* isolates by year and by country. FIN, Finland; FRA, France.

## Figures and Tables

**Figure A1:**
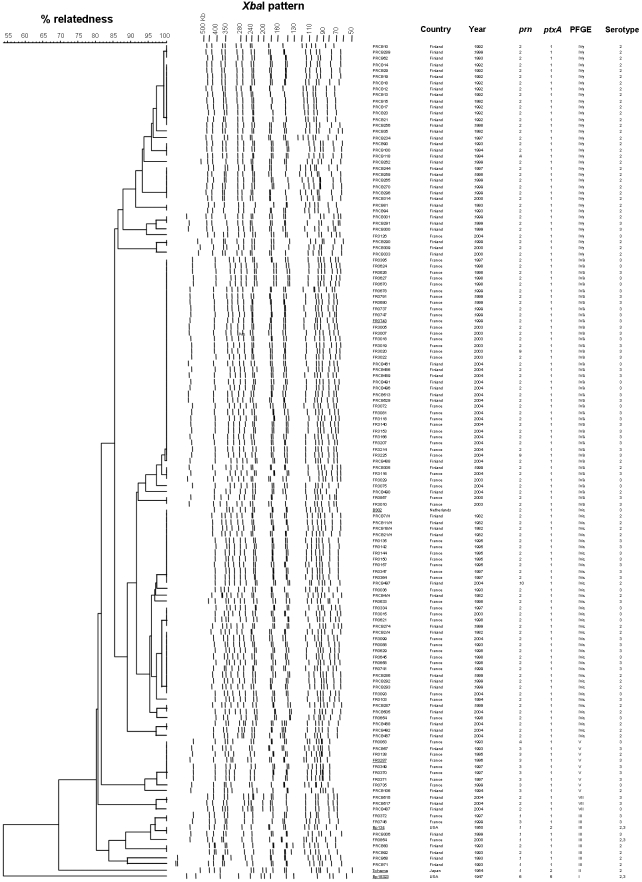
Genomic analysis of Finnish and French *Bordetella pertussis isolates*. From left to right, chromosomal DNA profiles. Download PDF
